# ASXL1 and DNMT3A mutation in a cytogenetically normal B3 thymoma

**DOI:** 10.1038/oncsis.2014.25

**Published:** 2014-07-07

**Authors:** R Belani, G Oliveira, G A Erikson, S Ra, M S Schechter, J K Lee, W J Shipman, S M Haaser, A Torkamani

**Affiliations:** 1 Medical Oncology Associates of San Diego, San Diego, CA, USA; 2 Scripps Genomic Medicine, The Scripps Translational Science Institute, Scripps Health and The Scripps Research Institute, San Diego, CA, USA; 3 San Diego Pathologists Medical Group, San Diego, CA, USA; 4 Imaging Healthcare Specialists, San Diego, CA, USA

## Abstract

The molecular drivers of thymoma are poorly understood. Outside of the identification of rarely occurring epidermal growth factor receptor and v-kit Hardy-Zuckerman 4 feline sarcoma viral oncogene homolog mutations via candidate gene sequencing, mutations in common cancer genes have yet to be observed. Only a single thymoma genome sequence has been previously reported, with no mutations in known cancer genes identified. Thus, we attempted to identify somatic driver mutations in a cytogenetically normal thymoma. A stage IVB type B3 thymoma from a 47-year-old male of Asian descent with no history of myasthenia gravis or other autoimmune condition was genomically evaluated. Exome sequencing and low-pass whole-genome sequencing was performed to identify somatic point mutations, copy number changes and structural variants. Mutations in known tumor suppressors DNMT3A (p.G728D) and ASXL1 (p.E657fs), consistent with mutations of known consequence in acute myeloid leukemia, were identified. Contrary to a previous report, this finding suggests the genetic etiology of thymomas may not be fundamentally distinct from other tumor types. Rather, these findings suggest that further sequencing of cytogenetically normal thymoma samples should reveal the specific molecular drivers of thymoma.

## Introduction

Thymomas are rare tumors arising from the epithelial cells of the thymus, commonly associated with myasthenia gravis or other autoimmune disorders.^1^ The WHO classification system categorizes thymomas (type A, B1, B2, B3 and AB) and thymic carcinoma (type C) according to morphology, degree of atypia, histological similarity to normal thymic cells and degree of lymphocyte infiltration.^2^ Thymomas are staged (stage I, IIA, IIB, III, IVA and IVB) according to degree of encapsulation and anatomic extent of disease with encapsulation decreasing and dispersion from the thymus increasing with stage.^3^ Late stage thymomas are very rare and tend to locally recur rather than metastasize. Type B3 thymomas are the most aggressive of the thymomas.

Surgical resection is the favored treatment for thymoma. Complete surgical resection can be achieved for the vast majority of stage I and II thymomas. Stage III–IV thymomas that cannot be fully resected are generally treated with radiation and platinum-based combination chemotherapy regimens leading to substantial tumor shrinkage;^4, 5, 6, 7^ however, similar survival is observed with preoperative radiation therapy without chemotherapy.^8, 9^ Currently, there are no good biomarkers for response to therapy. Amplifications and rare activating mutations of EGFR and overexpression of *HER2*, *KIT*, *BCL2* and *TP53*, as well as loss of *CDKN2A* have been previously described.^7, 10^ However, no strong evidence supports response of thymomas to EGFR or KIT targeted therapies.^11^

Thymomas and thymic carcinomas have been extensively profiled for chromosomal abnormalities, especially via comparative genomic hybridization,^12, 13, 14^ revealing that the vast majority of B3 thymomas display chromosomal abnormalities. However, there is no information on tumorigenic point mutations in B3 thymomas.^7^ Only a single genome sequence of a B3 thymoma has been reported thus far, for a tumor that displayed the typical chromosomal abnormalities for this tumor type (that is, gain of chromosome 1q, loss of chromosome 6 and 13q, and so on).^15^ No mutations in common cancer-associated genes were observed, leading the authors to suggest that thymomas may evolve through distinct molecular mechanisms as compared with other tumor types. In this article, we report the identification of *DNMT3A* and *ASXL1* mutations in a cytogenetically normal stage IVB type B3 thymoma. This is the first identification of driver point mutations in B3 thymoma and suggests that further genomic profiling of cytogenetically normal B3 thymomas may reveal the specific molecular drivers of this tumor type.

## Results

**Figure 1 fig1:**
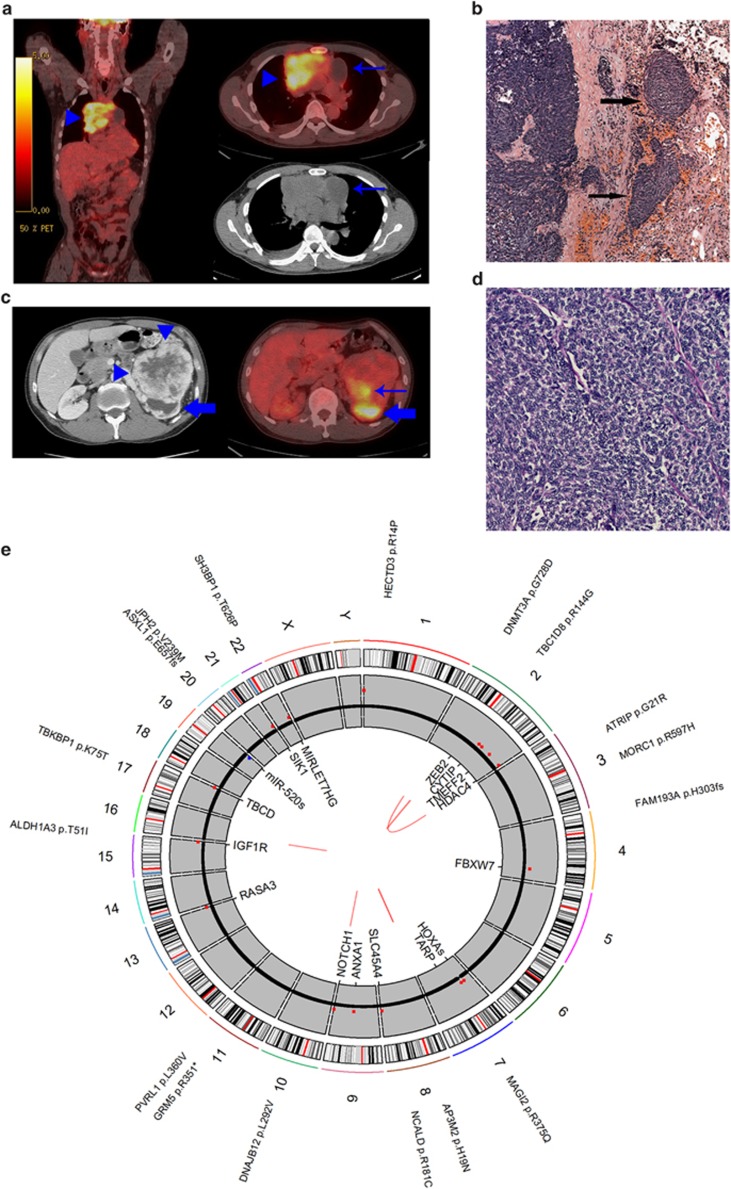
(**a**) Thymoma imaging. fluorodeoxyglucose positron emission tomography/computed tomography (CT) demonstrates intensely hypermetabolic anterior mediastinal mass on fused coronal and axial images (arrowheads). Cystic tumor component on attenuation-correction CT image is non-hypermetabolic on FDG PET (arrows). (**b**) Renal tumor imaging. CT scan with contrast (left) demonstrates large contrast-enhancing left renal mass (arrowheads) with posterior hydronephrotic upper pole collecting system (block arrow). Fused axial FDG PET/CT (right) demonstrates mildly hypermetabolic posterior tumor component (arrow). Upper pole hydronephrosis contains excreted urinary FDG (block arrow). (**c, d**) Thymoma histology. (**c**) Lobules of thymoma tumor cells infiltrate into the adjacent lung parenchyma (arrows: hematoxylin and eosin (H&E): × 5 magnification). (**d**) Sheets of thymoma type B3 polygonal tumor cells with ovoid nuclei (H&E: × 20 magnification). (**e**) Tumor genome overview. Circos plot overview of somatic mutations identified in the thymoma. Outer text denotes location and gene impacted by somatic protein coding mutations. Middle gray ring depicts copy number variation (CNV) status (black=2 copies, red=CNV gains, blue=CNV losses). Inner text displays genes overlapping identified CNVs. Inner red lines display identified structural variants.

The patient presented with a 3-month history of cough. He did not have shortness of breath, hemoptysis or weight loss at presentation. He also did not have neurologic symptoms consistent with myasthenia gravis. Family history is significant for thymoma in the mother. A computed tomography of the chest showed a mediastinal mass measuring 10.5 × 7.8 × 12.1 cm and a large (12 × 9.5 × 8.3 cm) left renal mass (Figures 1a and 1b, 1). The patient underwent computed tomography-guided biopsy of the mediastinal mass that revealed mildly atypical, uniform spindled cells associated with dense collagenous tissue. The tumor cells showed immunohistochemical positivity for AE1/AE3 and cytokeratin-7. Immunohistochemical markers for renal cell carcinoma were negative. The pathologic findings were consistent with thymoma. Subsequently, the patient underwent removal of mediastinal mass and right upper lobectomy as an en bloc resection. There was a separate right diaphragmatic nodule that was also excised. At the time of surgery, it was noted that the tumor was densely adherent to the pericardium and invaded the upper lobe of the right lung. Microscopic examination showed spindled to polygonal cells in varying sized lobules with accompanying fibrous bands of stroma invading the pericardium, pleura and lung (Figures 1c and d). Only occasional interspersed lymphocytes were seen. Focal lymph-vascular invasion was identified. The tumor cells showed ovoid nuclei with mild pleomorphism and scattered mitoses. The final margins were clear. The diaphragmatic nodule demonstrated tumor cells morphologically similar to the primary tumor. The final Masaoka stage of IVB was based on the finding of a separate distinct diaphragmatic tumor nodule and pleural invasion. The tumor had a WHO histologic category of B3 based on the predominance of epithelial cells and paucity of intraepithelial lymphocytes. No treatment was given before genome sequencing.

### Tumor genome overview

An overview of the somatic variants observed is presented in Figure 1e. Overall, 14 somatic indels and 176 somatic single-nucleotide polymorphisms were identified, 17 of which impacted the amino-acid sequence of proteins (Table 1). Sixteen copy number gains, 1 copy number loss (Table 2) and 6 structural variants (Table 3) were also observed. Sequencing of the renal mass revealed no overlap in somatic mutations, confirming the tumors were of independent origin. No major interchromosomal events were present in the thymoma genome, nor were any major chromosomal aneuploidies frequently observed in B3 thymomas.^12^ Structural variants that were detected did not contain breakpoints within genes of known relevance to tumorigenesis (Table 3). Thus, somatic events of interest were comprised of point mutations and subchromosomal copy number variants.

### Coding mutations

Fourteen nonsynonymous, 1 nonsense and 2 frameshift somatic mutations were observed in the tumor (Table 1). Of these mutations, four stood out with clear functional relevance—frameshift mutations in *FAM193A* and *ASXL1*, a nonsense mutation in *GRM5*, and a nonsynonymous mutation in *DNMT3A*. Although the mutations in FAM193A and GRM5 clearly impact protein function, the relevance of these mutations to tumorigenesis is not immediately apparent. On the other hand, the somatic mutations in *DNMT3A* and *ASXL1* are of the type previously observed in other tumor types and expected to contribute to tumorigenesis.

**Figure 2:**
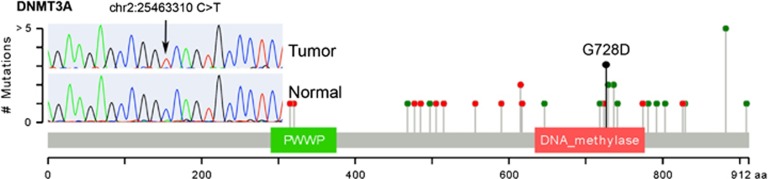
The DNMT3A p.G728D mutation observed in this thymoma sample is visualized in the context of other DNMT3A mutations observed in AML genome sequences from TCGA. The protein sequence and functional domains are depicted on the *x* axis. The number of AML mutations is depicted on the *y* axis. Red circles correspond to truncating mutations. Green circles correspond to missense mutations. Circle height corresponds to the number of mutations per position, however, the G728D indicator (black) is only meant to indicate position of this mutation. Note the clustering of AML nonsynonymous mutations around position 728. Mutations in this region reduce DNMT3A activity by disrupting the interaction between DNMT3L and DNMT3A. The Sanger sequencing validation trace of p.G7238D is also shown, demonstrating validation of p.G7238D as a homozygous somatic mutation.

DNMT3A is a DNA methyltransferase required for *de novo* methylation and mammalian development.^16^ It is known to act as a tumor suppressor in acute myeloid leukemia (AML) and is observed mutated less frequently in other tumor types.^17, 18^ The nonsynonymous mutation observed in this thymoma sample leads to a nonconservative substitution of aspartate for glycine at amino-acid 728 (p.G728D) within the DNA methylase domain of DNMT3A. This region is known to be responsible for the hydrophobic interaction between DNMT3A and its regulatory subunit DNMT3L, an interaction that is required for activation of DNMT3A methyltransferase activity and previously shown to be disrupted by mutations in this region.^19^ Moreover, previously observed nonsynonymous mutations in AML cluster tightly within this region (shown in Figure 2)^20, 21, 22^. Strikingly and unusually, this somatic mutation was observed in homozygous state in the tumor: 61 of 62 (98.4%) exome sequencing reads from the tumor supported the mutation, 0 of 41 (0%) of exome sequencing reads from the normal sample supported the mutation, the homozygous state was confirmed via Sanger sequencing (Figure 2, inset), and no evidence for loss of heterozygosity was observed in the exome or low-pass whole-genome sequencing data. Moreover, no other somatic mutations were observed at >60% allelic frequency in the tumor, suggesting a specific and early gene conversion event at this locus. Finally, a previously sequenced AML, reported by Hou *et al.*,^22^ carried a mutation at this position (p.G728R) and at a nearby position (p.F731L), supporting the notion that homozygous or compound heterozygous mutations at the DNMT3A—DNMT3L interface are required to effectively disrupt methyltransferase activity.^23^ Thus, the type, location and unusual zygosity of this somatic mutation strongly suggest mutations in *DNMT3A* have a causal role in the etiology of thymoma.

**Figure 3:**
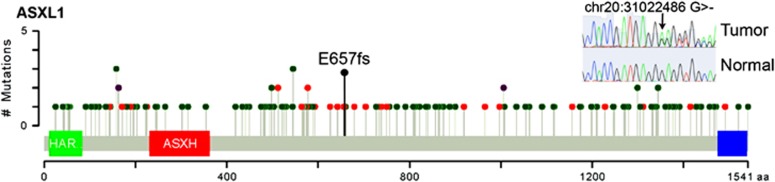
The ASXL1 p.E657fs mutation observed in this thymoma sample is visualized in the context of other ASXL1 mutations observed in all tumor genome sequences catalogued in TCGA. The protein sequence and functional domains are depicted on the *x* axis. The number of TCGA mutations is depicted on the *y* axis. Red circles correspond to truncating mutations. Green circles correspond to nonsynonymous mutations. Purple circles correspond to mutations that are both nonsynonymous and truncating in different gene isoforms. Circle height corresponds to the number of mutations per position, however, the E657fs indicator (black) is only meant to indicate position of this mutation. Note the clustering of TCGA truncating mutations around position 657. The Sanger sequencing validation trace of p.E657fs is also shown, demonstrating validation of p.E657fs as a heterozygous somatic mutation.

ASXL1 is a chromatin binding Polycomb group protein involved in transcriptional repression of numerous cell-type-specific systems, most likely through the recruitment of methylated histone H3 to promoters of target genes.^24^ *ASXL1* is a known tumor suppressor in myelodysplastic syndrome and chronic myelomonocytic leukemia although it is frequently mutated in other tumor types such as lung squamous and bladder cancer.^17, 18^ The heterozygous deletion of a single guanine nucleotide within exon 12 of *ASXL1* leads to out-of-frame translation beginning at codon 657 and premature truncation of the protein, removing >50% of the protein including the PHD domain responsible from histone interactions. This mutation was confirmed via Sanger sequencing (Figure 3, inset). Similar heterozygous C-terminal truncations are sufficient to induce myelodysplastic syndrome.^25^ Previously observed frameshift or nonsense mutations in *ASXL1* across numerous tumor types catalogued in The Cancer Genome Atlas (TCGA) have been observed both upstream and downstream of amino-acid 657, with the highest concentration centered at position 657 (Figure 3). In fact, the majority of previously observed *ASXL1* mutations occur in the same exon as this mutation, exon 12.^26, 27^ Thus, both the type and location of this somatic mutation strongly suggests mutations in *ASXL1* have a causal role in the etiology of thymomas.

### Copy number variants

A number of focal copy number gains were observed in this tumor, the most interesting being an amplification of the *HOXA* cluster (see Discussion section).^28^ Although a number of genes are known to be involved in tumorigenic processes are observed in these focal amplifications, no significance can be confidently assigned to these events.

## Discussion

Our results show that mutations in *DNMT3A* and *ASXL1* have a role in the development of thymoma. Our findings contrast starkly with a previous genome sequence from a B3 thymoma.^15^ However, the previously reported thymoma displayed cytogenetic abnormalities commonly observed in B3 thymomas, whereas this tumor displayed largely normal cytogenetics. These results are reminiscent of the preferential occurrence of driver point mutations in cytogenetically normal AML and suggests further sequencing of cytogenetically normal thymomas are fertile ground for the identification of the molecular drivers of thymic cancer.^29^ No obvious germline variant predisposing the patient to thymoma or renal cell cancer was observed, nor was there any overlap in somatic mutations between the thymoma and renal cell cancer to suggest shared etiology of these two tumors.

The first confidently identified driver mutation was a homozygous nonsynonymous mutation in *DNMT3A*. DNMT3A is a DNA methyltransferase essential for development, especially for *de novo* DNA methylation in stem cells.^16^ *DNMT3A* is mutated in numerous tumor types, but is mutated especially frequently in AML.^20, 21^ DNMT3A and its binding partner, DNMT3L form a tetramer: DNMT3L:DNMT3A:DNMT3A:DNMT3L.^23^ The most common DNMT3A mutations in AML occur at p.R882. Mutations at this position disrupt binding at the DNMT3A:DNMT3A interface, and impact activity in dominant-negative manner,^23, 30^ or at least reduce the odds of proper tetramer assembly by ∼75% as a single mutated DNMT3A protein is sufficient to disrupt tetramer assembly. Alternatively, the DNMT3L:DNMT3A interface is disrupted by somatic mutations within the mutational hotspot region centered on the p.G728D mutation observed in this thymoma.^23^ Heterozygous mutations at this site may not be expected to influence activity as markedly as mutations at p.R882 as they likely allow for the formation of partially complete complexes, for example, DNMT3A:DNMT3A:DNMT3L complexes could form 50% of the time. Thus, it is notable that the p.G728D mutation was observed in homozygous state in this tumor. Homozygous somatic mutations are highly unusual, suggesting DNA repair via double-stranded break repair or synthesis-dependent strand annealing and/or some other gene conversion event occurred specifically at this site given we did not find any evidence for loss of heterozygosity.

The second confidently identified driver mutation was a frameshift mutation in exon 12 of *ASXL1*. ASXL1 is a known tumor suppressor in hematological malignancies, where the majority of frameshift and nonsense mutations are also observed in exon 12.^26, 27^ ASXL1 loss leads to loss of H3K27me3 and increased expression of genes repressed by these histone marks, especially the anterior HOXA gene cluster.^24^ HOXA genes themselves are capable of leukemogenic transformation.^31^ Interestingly, we also observe a concomitant and specific amplification of the HOXA gene cluster in this tumor.

It is intriguing that both *DNMT3A* and *ASXL1* are known tumor suppressors in hematological malignancies^18^ and that the observed mutation characteristics are consistent with those seen in AML. However, both genes have been observed mutated, albeit at lower frequencies, in epithelial malignancies as well. Although co-occurrence of *DNMT3A* and *ASXL1* mutations are quite rare in epithelial and hematological malignancies, sequencing of a larger number of thymomas will be required to determine whether this co-occurrence is a happenstance of our subject selection or whether it points to a specific requirement of marked epigenetic reprogramming in the neoplastic transformation of thymic cells. The extent of *DNMT3A* and *ASXL1* mutations in thymoma should be explored, and treatments targeting epigenetic reprogramming should be considered in the future.

## Materials and methods

### Study consent

The study participant provided written informed consent under a protocol approved by the institutional review board of Scripps.

### Tumor and normal sample

The formalin-fixed, paraffin-embedded block containing the thymoma tumor was sectioned 11 times, with each section placed on a separate slide. One section, at 4-μm thick, was stained and used to assess tumor area and cellularity. The boundaries of the tumor were marked, and the tumor cellularity was estimated to be >85%. The additional 10 sections were cut at 10-μm thicknesses, and the designated (the boundaries were traced onto each slide using the stained and marked slide) tumor areas were extracted and isolated using the RecoverAll FFPE nucleic acid isolation kit (Ambion, Austin, TX, USA). Freshly drawn blood was obtained for the analysis of normal DNA. Genomic DNA was extracted from whole blood using the QIAamp system (Qiagen, Valencia, CA, USA). Sequence alignment and variant calling were performed against the reference human genome (NCBI 37/hg 19).

### Whole-exome sequencing, variant calling and filtration

Whole-exome sequencing was pursued utilizing Agilent SureSelect exome hybridization followed by barcoding and sequencing of paired 100-bp reads on the equivalent of ∼1 lane of an Illumina HiSeq2500 (Illumina, San Diego, CA, USA) instrument (samples were barcoded and distributed across multiple lanes). Read mapping and initial variant calling and quality filtration was performed using the standard BWA-GATK^32, 33^ best practices variant quality score recalibration approach. A mean coverage of 157.3X and 98.7X was achieved for the tumor and normal sample with 96.1% and 98.7% of the target exome covered by >10 reads, respectively.

Somatic point mutations were detected using MUTECT.^13^ Somatic indels were detected using SomaticIndelDetector.^32^ Somatic variants were then annotated using the SG-ADVISER (genomics.scripps.edu/ADVISER/) and Cypher Genomics, Inc. (San Diego, CA, USA) systems for further filtration. Somatic variants present at >0.1% frequency in the HapMap,^14^ 1000 Genomes,^34^ NHLBI GO Exome Sequencing Project,^35^ or 385 genomes from the Scripps Wellderly population, as well as variants present in segmental duplication regions that are prone to produce false-positive variant calls were removed. Somatic indels were stringently filtered to remove indels within simple repeats, repeats identical to the inserted/deleted sequence or adjacent to homopolymer repeats of length 5+. Nonsynonymous, in-frame, frameshift, nonsense or canonical splice-site donor/acceptor site variants were retained for further analysis.

### Low-pass whole-genome sequencing, structural variant calling and filtration

Low-pass whole-genome sequencing was performed by barcoding and sequencing of paired 100-bp reads on the equivalent of ∼1 lane of an Illumina HiSeq2500. A mean coverage of 7.4X and 7.7X was achieved for the tumor and normal sample with 92.0% and 95.5% of the genome covered by >3 reads, respectively. Copy number and structural variants were identified by SVDetect.^36^ For copy number variants, read density was determined for 100-kb windows at 10-kb intervals. The significance of read density differences between the tumor and normal sample were determined using a binomial test with the normal sample determining the probability of read mapping per window in order to adjust for mapability and other window-specific effects. Significant windows (*P*-value threshold=1E–8) were stringently filtered to retain called copy number variations only where the ratio of tumor to normal reads, adjusted for the difference in total number of reads, was at least 1.5X greater for copy number gains or 0.5X lower for copy number losses. For structural variants, an initial set of raw candidate structural variants was called using SVDetect with at least seven reads supporting structural variant calls in the tumor sample and three reads supporting calls in the normal sample. Tumor sample candidate structural variants were then filtered to remove structural variants with support in the normal sample or mapping to centromeric regions.

Circos plots were generated via RCircos.^37^ Mutation plots were generated via adaption of cBioPortal visualization plots.^38^

## Figures and Tables

**Table 1:** Coding mutations

*Gene*	*Gene impact*	*Protein location*	*Prediction of impact*	*Coordinates (hg19)*
ALDH1A3	Missense	T51I	SIFT, Condel	chr15: 101 425 524 snp C>T
AP3M2	Missense	H19N	Polyphen, SIFT, Condel	chr8: 42 012 260 snp C>A
ASXL1	Frameshift	E657fs	Damaging	chr20: 31 022 486 del G>-
ATRIP	Missense	G21R	SIFT, Condel	chr3: 48 488 310 snp G>C
DNAJB12	Missense	L292V	Neutral	chr10: 74 098 021 snp G>C
DNMT3A	Missense	G728D	Polyphen, SIFT, Condel	chr2: 25 463 310 snp C>T
FAM193A	Frameshift	H303fs	Damaging	chr4: 2 648 428 ins ->T
GRM5	Nonsense	R351*	Damaging	chr11: 88 386 432 snp G>A
HECTD3	Missense	R14P	SIFT, Condel	chr1: 45 476 889 snp C>G
JPH2	Missense	V239M	Neutral	chr20: 42 788 712 snp C>T
MAGI2	Missense	R375Q	Condel	chr7: 77 807 386 snp C>T
MORC1	Missense	R597H	Neutral	chr3: 108 724 077 snp C>T
NCALD	Missense	R181C	Polyphen, SIFT, Condel	chr8: 102 701 578 snp G>A
PVRL1	Missense	L360V	Neutral	chr11: 119 510 648 snp G>C
SH3BP1	Missense	T626P	Neutral	chr22: 38 051 466 snp A>C
TBC1D8	Missense	R144G	Neutral	chr2: 101 670 771 snp T>C
TBKBP1	Missense	K75T	Polyphen, SIFT, Condel	chr17: 45 773 702 snp A>C

Somatic protein coding mutations.

**Table 2:** Copy number variants

*Type*	*Coordinates*	*Size (kb)*	*Dosage*	*Overlapping genes*
Gain	chr1: 470 001–600 000	130	5	None
Gain	chr2: 145 130 001–145 310 000	180	3	ZEB2
Gain	chr2: 158 260 001–158 370 000	110	3	CYTIP
Gain	chr2: 192 770 001–192 940 000	170	3	TMEFF2
Gain	chr2: 240 110 001–240 240 000	130	3	HDAC4
Gain	chr4: 153 220 001–153 320 000	100	3	FBXW7
Gain	chr7: 27 120 001–27 240 000	120	3	HOXA gene cluster, MIR196B
Gain	chr7: 38 290 001–38 390 000	100	3	TARP
Gain	chr8: 142 200 001–142 320 000	120	3	SLC45A4, DENND3
Gain	chr9: 75 690 001–75 830 000	140	3	ANXA1, ALDH1A1
Gain	chr9: 139 420 001–139 520 000	100	3	NOTCH1, MIR4674
Gain	chr13: 114 740 001–114 920 000	180	3	RASA3
Gain	chr15: 99 410 001–99 510 000	100	3	IGF1R
Gain	chr17: 80 740 001–80 920 000	180	3	TBCD, ZNF750, B3GNTL1
Loss	chr19: 54 070 001–54 200 000	130	1	ZNF331, DPRX, MIR520 cluster
Gain	chr21: 44 690 001–44 900 000	210	3	SIK1
Gain	chr22: 46 380 001–46 550 000	170	3	MIRLET7B, MIR3619, MIR4763

Tumor-specific copy number variants, dosage and contained genes.

**Table 3:** Structural variants

*Type*	*State*	*Breakpoint 1*	*Breakpoint 2*	*Overlapping genes*
Coamplicon	Bal	chr16: 26 382 735–26 386 932	chr16: 26 382 736–26 386 932	None
Inversion	Unbal	chr2: 87 651 839–87 656 090	chr2: 90 482 288–90 485 529	Many
Inversion	Unbal	chr2: 33 141 477–33 141 679	chr2: 242 819 853–242 820 060	Many
Coamplicon	Bal	chr7: 158 706 424–158 710 034	chr7: 158 706 425–158 710 035	WDR60
Insertion	Bal	chr7: 158 704 999–158 708 434	chr7: 158 705 000–158 709 994	WDR60
Insertion	Bal	chr9: 139 994 967–139 997 831	chr9: 139 994 968–139 998 202	MAN1B1

Abbreviations: Bal, balanced; Unbal, unbalanced.

Tumor-specific structural variants.

## Notes

Dr Belani declares an ownership interest in the Medical Oncology Associates of San Diego, intellectual property ownership with Atyr Pharma and is an adviser for Dendreon and Bayer. Dr Torkamani declares he is a co-founder and equity owner of Cypher Genomics, Inc. The remaining authors declare no conflict of interest.
